# α-Synuclein in the Synaptic Vesicle Liquid Phase: Active Player or Passive Bystander?

**DOI:** 10.3389/fmolb.2022.891508

**Published:** 2022-05-18

**Authors:** Lennart Brodin, Dragomir Milovanovic, Silvio O. Rizzoli, Oleg Shupliakov

**Affiliations:** ^1^ Department of Neuroscience, Karolinska Institutet, Stockholm, Sweden; ^2^ Laboratory of Molecular Neuroscience, German Center for Neurodegenerative Diseases (DZNE), Berlin, Germany; ^3^ Institute of Neuro- and Sensory Physiology, University Medical Center Göttingen, Göttingen, Germany; ^4^ Institute of Translational Biomedicine, St. Petersburg University, St. Petersburg, Russia

**Keywords:** synapse, synaptic vesicle, liquid-liquid phase separation (LLPS), synaptic vesicle clustering, α-Synuclein, synapsin

## Abstract

The protein α-synuclein, which is well-known for its links to Parkinson’s Disease, is associated with synaptic vesicles (SVs) in nerve terminals. Despite intensive studies, its precise physiological function remains elusive. Accumulating evidence indicates that liquid-liquid phase separation takes part in the assembly and/or maintenance of different synaptic compartments. The current review discusses recent data suggesting α-synuclein as a component of the SV liquid phase. We also consider possible implications of these data for disease.

## Introduction

**FIGURE 1:**
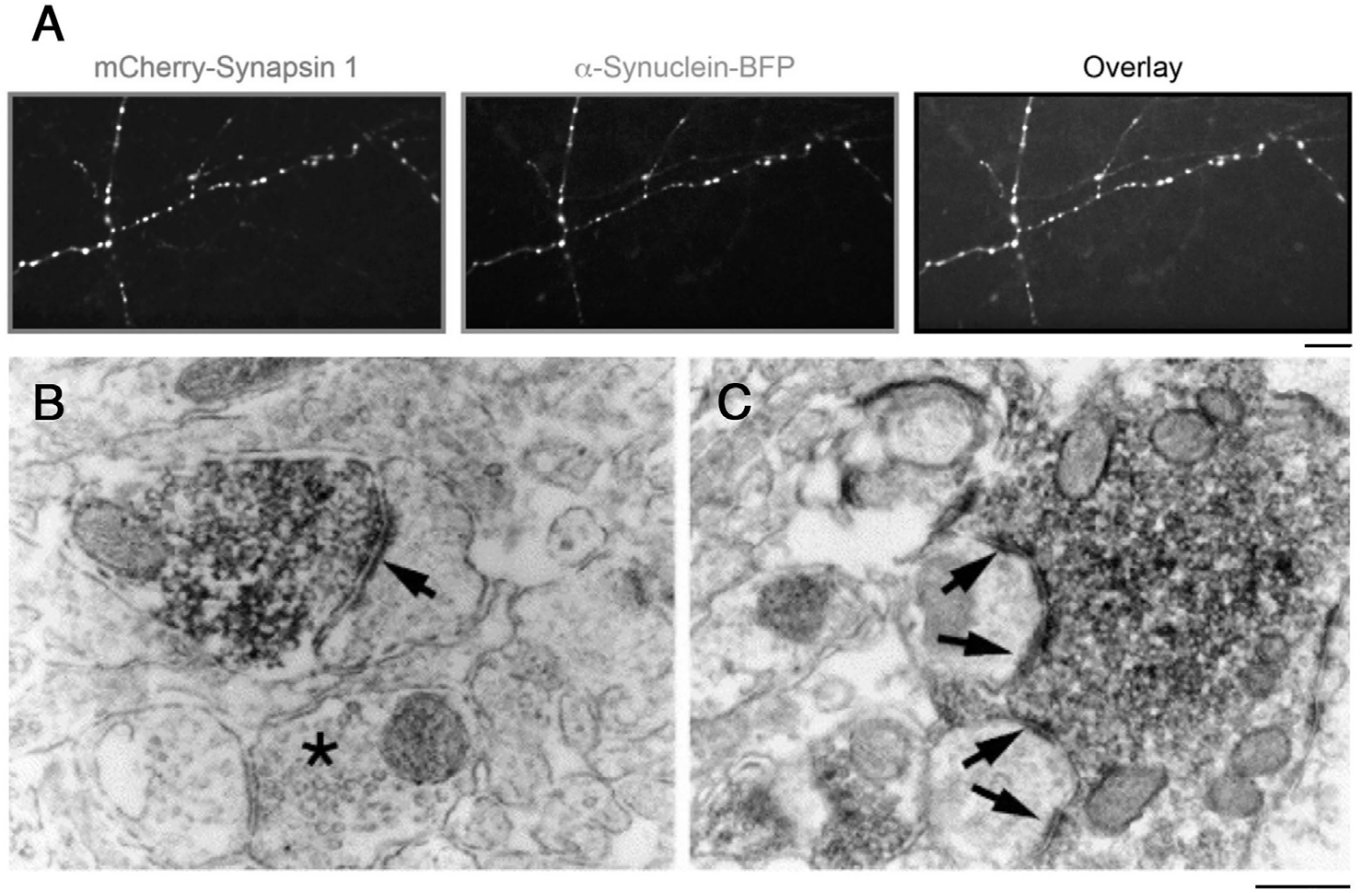
Accumulation of α-synuclein and synapsin in SV clusters. **(A)** mCherry-synapsin I and α-synuclein-BFP constructs are targeted to synapses in primary hippocampal neurons. Heterologous expression of mCherry-Synapsin I. Scale: 10 µm. From (Hoffmann et al., 2021). **(B,C)** Electron microscopic images of symmetrical synaptic contacts (arrows) in the prefrontal cortex **(B)** and hilus **(C)** labeled with anti-α-synuclein antibodies. Note an accumulation of the synuclein labeling over synaptic vesicle clusters. Scale: 0.3 µm. From (Totterdell et al., 2004).

The first synuclein protein was identified by its association with synaptic vesicles (SVs) in the *Torpedo* electric organ (Maroteaux et al., 1988). Subsequently, three homologous members were identified in mammals, α-, β-, and γ-synuclein, and the enrichment of the latter in synaptic boutons and SV clusters in mammalian neurons was confirmed [Figure 1; (Totterdell et al., 2004; Hoffmann et al., 2021)]. α-Synuclein has been the focus of intense research efforts due to its strong coupling with Parkinson’s disease (PD) and other synucleinopathies: it is a component of the Lewy bodies occurring in the diseased brain, and mutations in α-synuclein cause rare familial variants of PD (Simon et al., 2020).

α-Synuclein is natively unfolded in solution but adopts an α-helical conformation in contact with acidic phospholipids and/or highly curved phospholipid membranes (Sulzer and Edwards, 2019). This change involves the formation of so-called ALPS-motifs (amphipathic lipid packing sensor motifs), which can bind to and stabilize curved membranes (Antonny, 2011; Pranke et al., 2011; Westphal and Chandra, 2013; Runwal and Edwards, 2021). Moreover, the C-terminal part can bind phospholipids in a calcium-dependent manner (Lautenschlager et al., 2018). These properties of α-synuclein are likely to explain its strong association with SVs. Only low levels of α-synuclein occurs in the cytosol of neurons (Gerdes et al., 2020).

The enrichment of α-synuclein in the SV cluster of synapses is suggestive of a physiological role in the SV cycle. However, synucleins are not essential for neurotransmitter release. No discernable homologs have been detected in invertebrates, and triple knockout of synucleins in mice only gives rise to subtle changes of synaptic function (Bendor et al., 2013; Runwal and Edwards, 2021). Mice lacking α-synuclein appear essentially normal and have a normal lifespan. Thus, in the context of synapse physiology, α-synuclein appears to play redundant roles. Several links of α-synuclein to presynaptic functions have nonetheless been suggested. SV-bound α-synuclein dissociates upon exocytosis and subsequently re-associates, consistent with an involvement in SV cycling (Fortin et al., 2005). Overexpression and knockout studies point to roles of α-synuclein in different steps of the SV cycle. These include control of the fusion pore (Logan et al., 2017), regulation of SNARE proteins (Burre et al., 2010), involvement in SV recycling (Vargas et al., 2014), and in the regulation of axonal transport of SVs (Scott and Roy, 2012). In addition, a function in mitochondrial dynamics has been proposed (Kamp et al., 2010; Nakamura et al., 2011; Guardia-Laguarta et al., 2014). These putative functions have recently been the subject of insightful reviews (Sulzer and Edwards, 2019; Runwal and Edwards, 2021) and will therefore not be further considered here. Instead, we will focus on the possible involvement of α-synuclein in the organization of the SV cluster by liquid-liquid phase separation (LLPS), it’s possible roles as a part of this liquid phase, and factors contributing to the formation of pathological protein aggregates.

## Phase Separation in the Nerve Terminal

The nerve terminal in excitatory synapses contains three structurally distinct compartments, the active zone (AZ), the proximal portion of the SV cluster (near the AZ), and the distal portion of this cluster. The AZ comprises a dense proteinaceous matrix, which covers the presynaptic plasma membrane and is aligned with the postsynaptic density. It contains calcium channels and numerous proteins, many of which contain large helical regions with molecular weights exceeding 400 kDa (Sudhof, 2012; Gundelfinger et al., 2015; Acuna et al., 2016). In direct apposition to the AZ lies the vesicle pool that comprises about 3–4 layers of SVs and is distinguished by its independence of synapsin at rest (Pieribone et al., 1995; Rosahl et al., 1995; Siksou et al., 2007; Vasileva et al., 2012). This pool, here referred to as the proximal SV pool, is not evident in inhibitory synapses (Gitler et al., 2004). On the top of the proximal pool lies a large distal pool that comprises the bulk of the SV cluster. The distal pool is disrupted by knockout or perturbations of synapsin (Pieribone et al., 1995; Rosahl et al., 1995; Siksou et al., 2007; Vasileva et al., 2012). While SV pools have also been defined by physiological criteria (Denker and Rizzoli, 2010; Chanaday and Kavalali, 2018), it is important to note that such functional pools do not correlate with the two structural SV pools discussed here.

In the context of cell biology, LLPS is an emerging principle of subcellular organization by which biomolecules, including proteins, nucleic acids and vesicles, form mesoscale assemblies through multivalent, low-affinity interactions (Banani et al., 2016; Shin and Brangwynne, 2017). These interactions can be mediated by intrinsically disordered regions (IDRs) of proteins or modular binding domains, such as SH3 domains binding proline-rich motifs, allowing for protein/protein, protein/membrane or protein/nucleic acid interactions (Li et al., 2012; Banani et al., 2016; Shin and Brangwynne, 2017; Mittag and Parker, 2018; Dignon et al., 2020; Zhao and Zhang, 2020).

Recently, LLPS has been implicated in the assembly and/or maintenance of two presynaptic compartments, the AZ and the distal part of the SV cluster. *In vivo* studies in *Caenorhabditis elegans* showed that the assembly of the developing AZ depends on phase separation of the core AZ proteins SYD-2 and ELKS-1 (McDonald et al., 2020). Notably, synaptic SYD-2/ELKS-1 condensates remain in a liquid state only during early developmental stages but mature into hydrogel-like structures at later stages (McDonald et al., 2020). Corresponding *in vivo* experiments have not yet been performed in vertebrates, but *in vitro* studies have shown that the AZ proteins RIM and RIM-BP can undergo LLPS *in vitro* (Wu et al., 2019). Such RIM/RIM-BP condensates can recruit calcium channels, consistent with a role in AZ assembly (Wu et al., 2019).

Regarding the SV cluster, pioneering *in vitro* studies pointed at synapsin as an organizer by means of LLPS. Synapsin or its isolated IDR can form protein droplets in solution (Milovanovic et al., 2018), either alone or together with small acidic liposomes (Milovanovic et al., 2018), or with isolated SVs (Hoffmann et al., 2021). These *in vitro* data are supported by *in vivo* observations (Milovanovic and De Camilli, 2017). Unlike the active zone, however, the SV cluster appears to remain in a liquid state in the adult nervous system. Thus, SV clusters in mature synapses exhibit dynamic properties compatible with a liquid phase (Betz et al., 1992; Kamin et al., 2010; Staras et al., 2010; Milovanovic and De Camilli, 2017). Moreover, the acute disruption of interactions of the synapsin IDR in an adult synapse causes dispersal of the distal SV pool (Pechstein et al., 2020).

While numerous membrane-less organelles have been shown to be organized by LLPS the vast majority of these contain only proteins and/or proteins and RNA. The SV cluster, together with assemblies of COPII vesicles (Zhao and Zhang, 2020), is thus distinct from most other membrane-less organelles in that it contains lipid vesicles.

In addition to synapsin and α-synuclein, the SV cluster contains a plethora of proteins [Table 1; (Denker et al., 2011; Shupliakov, 2009)], many of which bind to SVs (Perego et al., 2020; Reshetniak et al., 2020). The majority of these proteins contain IDR(s) and some contain SH3 domains (Table 1). The possible roles of these proteins in the maintenance of the SV liquid phase is presently unclear (regarding α-synuclein, see below). Regarding the SH3 domain-containing proteins it is interesting to note that the SH3 domain pentamer of intersectin co-assembles with synapsin in protein droplets and can stimulate their formation (Milovanovic et al., 2018). In view of the well-established role of SH3 domain multimers in LLPS (Li et al., 2012; Banani et al., 2016; Ghosh et al., 2019) it is thus possible that intersectin promotes phase separation in the SV cluster. On the other hand, it is unclear to what an extent intersectin acts as a multivalent binding partner of synapsin as only one of its SH3 domains (SH3A) shows detectable synapsin binding (Gerth et al., 2017; Pechstein et al., 2020). Moreover, in the resting intact synapse, antibodies inhibiting interactions with the SH3A domain does not interfer with SV clustering, nor does antibodies inhibiting interactions with the amphiphysin SH3 domain (Pechstein et al., 2020). These observations do, however, not rule out a combined contribution of several cluster-enriched SH3 domain proteins on phase separation of SVs. Would there be such a contribution, it can be anticipated that it is under complex regulation by the many components of the SV cluster (Ghosh et al., 2019).

**TABLE 1:** Properties of proteins enriched in the synaptic vesicle cluster.

Protein	IDR	SH3 domain	Fold enrichment in SV cluster versus rest of synapse	Primary role	References
α- SNAP	−	−	35	Post exocytosis	Reshetniak et al. (2020)
α-synuclein	+	−	37	Unclear	Maroteaux et al. (1988)
Amphiphysin	++	+	40	Endocytosis	Evergren et al. (2004)
AP180	++	−	27	Endocytosis	Denker et al. (2011)
Calmodulin	−	−	8	Ca^2+^ binding	Reshetniak et al. (2020)
Clathrin	−	−	11	Endocytosis	Denker et al. (2011)
Complexin	−	−	19–34	Exocytosis	Denker et al. (2011)
Doc2a	+	−	29	Endocytosis	Reshetniak et al. (2020)
Dynamin	++	−	86	Endocytosis	(Evergren et al., 2007; Denker et al., 2011)
EHD	−	−	Not studied	Endocytosis	Jakobsson et al. (2011)
Endophilin	+	+	19	Endocytosis, autophagy	(Shupliakov, 2009; Bai et al., 2010; Sundborger et al., 2011)
Eps15	++	−	Not studied	Endocytosis	Koh et al. (2007)
Epsin	++	−	44	Endocytosis	Jakobsson et al. (2011)
Hsc70	+	−	8	Chaperone	Denker et al. (2011)
Intersectin	++	+++++	90	Endocytosis	Evergren et al. (2007)
Munc13	++	−	19	Exocytosis	Reshetniak et al. (2020)
Munc18	−	−	27	Exocytosis	Reshetniak et al. (2020)
NSF	−	−	11	Post exocytosis	Denker et al. (2011)
Rab3	+	−	19	Exocytosis	Fischer von Mollard et al. (1990)
Rab5	++	−	19	Endocytosis	Reshetniak et al. (2020)
Rabphilin	++	−	Not studied	Exocytosis	Denker et al. (2011)
RIM	++	−	Not studied	Exocytosis	Denker et al. (2011)
SCAMP1	++	−	Not studied	Carrier	Reshetniak et al. (2020)
Septin5	−	−	19	Nucleotide binding, autophagy	Reshetniak et al. (2020)
Synapsin	++	−	134	SV clustering	De Camilli et al. (1983)
Syndapin	++	+	35	Endocytosis	Andersson et al. (2008)
Synaptojanin	++	−	Not studied	Endocytosis	McPherson et al. (1996)

Features of soluble proteins enriched in synaptic vesicle clusters. For intrinsically disordered regions (IDRs): + = a stretch of >20 amino acids with a disorder probability of >0.6; ++ = a stretch of >50 amino acids with a disorder probability of >0.6 according to PrDOS (Ishida and Kinoshita, 2007). Values were calculated on the human 1/A isoform for each protein. For SH3 domains: each + indicates one SH3 domain.

A growing field of study regard interactions between intrinsically disordered proteins and membranes (Zhao and Zhang, 2020; Hicks et al., 2021). How do such interactions pertain to the SV cluster liquid phase? It is first important to note that the SV liquid phase, confined to the distal portion of the SV cluster, is not in contact with the plasma membrane as the two are spatially separated by the proximal portion of the cluster. Regarding SVs, the multiplicity of interactions with cluster-enriched proteins (Perego et al., 2020; Reshetniak et al., 2020) may well involve IDR-membrane interactions. For example, synapsin primarily binds SVs *via* an N-terminal region (Benfenati et al., 1989; Krabben et al., 2011), but an additional contribution of its IDR cannot be excluded. Likewise, many of the proteins listed in Table 1 may potentially interact with SVs *via* their IDRs. Hence, the SV membrane may serve as an additional template for LLPS.

It remains to be determined how the proximal SV pool is organized. The protein matrix of the AZ extends, at least partly, into this pool (Sudhof, 2012; Gundelfinger et al., 2015). However, liquid condensates formed by RIM and RIM-BP do not incorporate SVs, but only absorb them on their surface (Wu et al., 2021). It seems likely that the protein matrix and vesicles at the AZ are creating a surface that favors the attachment of the SV phase of the distal pool.

Apart from organizing the distal vesicle pool, LLPS may take part in SV endocytosis in the periactive zone, as LLPS has been implicated in other forms of clathrin-mediated endocytosis (Schiano Lomoriello et al., 2022). Notably, however, specific data on endocytosis in the synapse is presently lacking.

## Possible Involvement of α-Synuclein in Organizing the Synaptic Vesicle Liquid Phase

**FIGURE 2:**
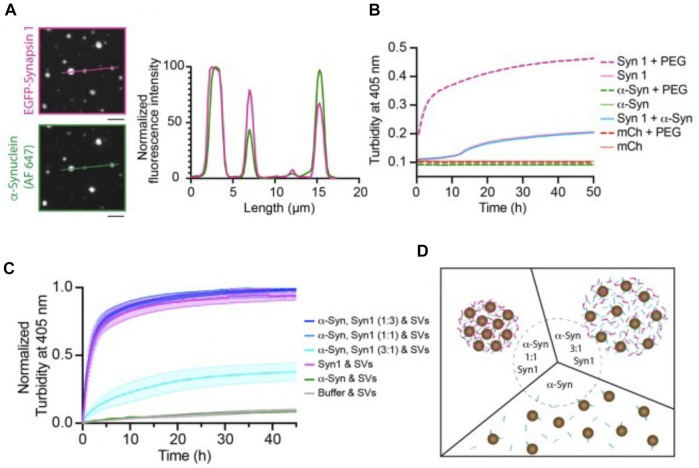
Synapsin recruits α-synuclein into liquid protein droplets but α-synuclein is not able to drive phase separation effectively and recruit synaptic vesicles into the liquid droplets. **(A)** Colocalization of reconstituted condensates containing EGFP-Synapsin 1 (6 µM) and α-synuclein (2 mM, chemically labeled with Alexa Fluor 647, AF 647) in 3% PEG, 8,000. Scale bars, 5 µm. **(B)** Condensate formation of purified recombinant proteins. 6 µM EGFP-synapsin one in magenta; 6 µM mCherry in red; 2 µM α-synuclein in green in the absence (full line) or presence of 3% PEG 8000 (dashed line). The condensate formation was measured as a change in turbidity at 405 nm. **(C)** Excess of α-synuclein reduces the rate of synapsin condensate formation, and α-synuclein is unable to recruit SVs. Condensate formation of purified recombinant proteins EGFP-synapsin I and α-synuclein in different molar ratios (curves in tones of blue), EGFP-synapsin one alone (magenta), α-synuclein alone (green) in presence of 23 nM SVs. The condensate formation was measured as a change in turbidity at 405 nm. Each value is shown as the average ±standard error of the mean, data are from three independent replicates (each time fresh isolation of native SVs). **(D)** Scheme of the synapsin/SV condensation in the presence of different molar ratios of α-synuclein (α-synuclein-to-synapsin, 1:1 left and 3:1 right) or in the absence of synapsin (bottom). From (Hoffmann et al., 2021).

To what extent may α-synuclein contribute to the organization of the SV liquid phase? While both synapsin and α-synuclein have IDRs, recent *in vitro* studies do not favor a role of α-synuclein as an initiator of the SV liquid phase. For example, α-synuclein does not undergo LLPS *in vitro* at physiological (i.e., low micromolar) concentrations, which contrasts with the behavior of synapsin [Figure 2B; (Hoffmann et al., 2021)]. Moreover, α-synuclein cannot recruit isolated SVs into protein droplets [Figures 2C,D; (Hoffmann et al., 2021)]. Together with the fact that SV clusters persist in synuclein triple knockout mice (Vargas et al., 2017), these data argue against a role of α-synuclein as an initiator of the SV liquid phase. Nevertheless, α-synuclein may be one of several factors that contribute to maintaining the SV liquid phase. In fact, all proteins listed in Table 1 that contain IDRs and/or SH3 domains may potentially fulfill this role. Additionally, integral SV proteins may contribute (Kim et al., 2021; Park et al., 2021).

Interestingly, recent *in vitro* experiments have shown that α-synuclein at low micromolar concentrations can be recruited into synapsin droplets and it remains in a liquid state therein [Figure 2A; (Hoffmann et al., 2021)]. A role of α-synuclein in contributing to the SV liquid phase is consistent with the observation that α-synuclein multimers can cluster vesicles and restrict their motility under *in vitro* conditions (Pranke et al., 2011). It is also consistent with ultrastructural studies showing that SV clusters have a higher packing density in synuclein knockout mice than in control animals (Vargas et al., 2017). Moreover, the lamprey synuclein ortholog has very high homology to mammalian synuclein (Busch et al., 2014; Vorontsova et al., 2018; Fouke et al., 2021), and microinjection of pan-synuclein antibodies into the giant reticulospinal axon resulted in migration of SVs away from the SV cluster in a piecemeal fashion (i.e., small packets of vesicles), suggesting the putative role of α-synuclein in higher-level assembly of SV condensates (Fouke et al., 2021).

## Can α-Synuclein Alone Undergo Phase Separation Under Physiological Conditions?

**FIGURE 3:**
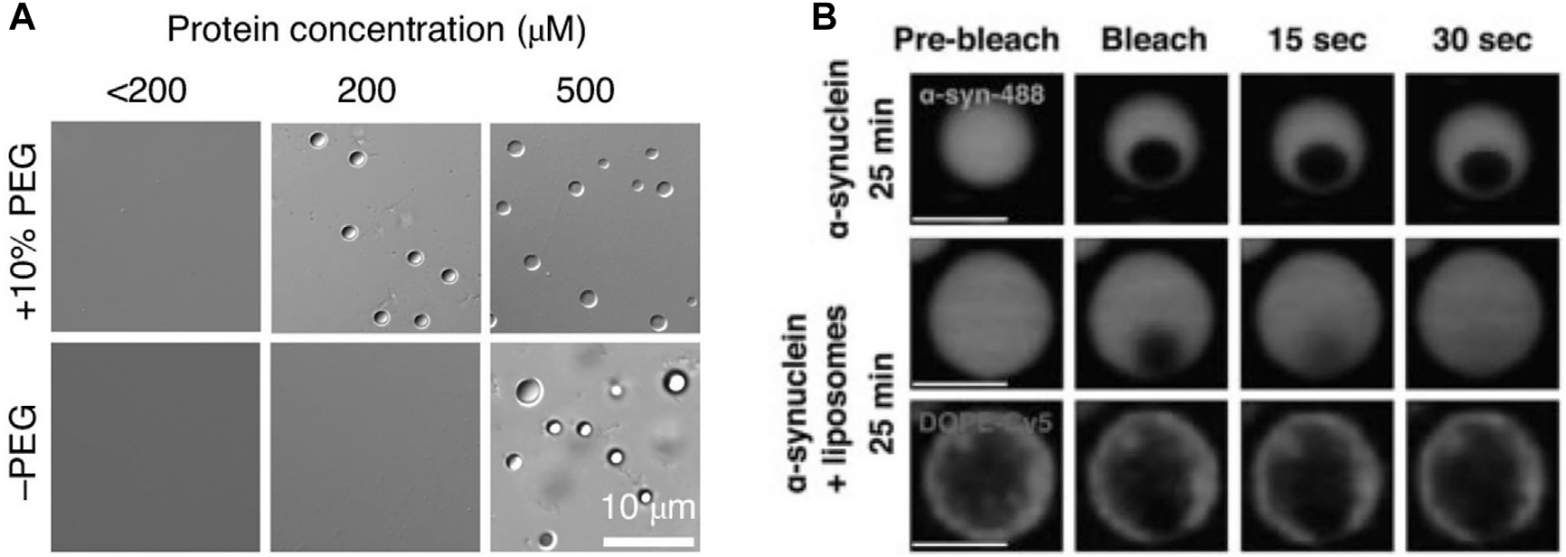
α-synuclein can undergo liquid-to-solid transition at high concentrations *in vitro*, which is reduced by negatively charged liposomes. **(A)** α-Synuclein (α-Syn) undergoes LLPS *in vitro*. Differential interference contrast (DIC) images of α-Syn phase-separated droplets at different protein concentrations in the presence and absence of the molecular crowder PEG-8,000. From (Ray et al., 2020). **(B)** α-synuclein/liposome droplets mature slower than droplets containing α-synuclein only (protein:lipid, 1:1). Lowering the protein:lipid ratio to 10:1 results in a loss of the protective effect. From (Hardenberg et al., 2021).

At high concentrations (i.e., 200 µM) α-synuclein alone can undergo LLPS *in vitro* (Figure 3A; (Ray et al., 2020). Importantly, under these conditions, there is also a maturation of α-synuclein droplets from a liquid into a solid-like state (Figure 3B) (Hardenberg et al., 2021; Ray et al., 2020). A similar maturation occurs when α-synuclein is ectopically overexpressed in cells (Hardenberg et al., 2021). How might these findings relate to *in vivo* conditions? The precise concentration of α-synuclein in the SV cluster is not known. The average concentration in nerve terminals is in the 20 µM range, based on a measured concentration of α- and β-synuclein of 43 µM in synaptosomes (Wilhelm et al., 2014). The concentration of α-synuclein in SV clusters is probably several-fold higher due to its efficient binding to the curved SV membrane, with the concentration of free α-synuclein in the synapse cytosol, away from the vesicle clusters, being estimated to only ∼1.2 µM (Reshetniak et al., 2020). Interactions with other cluster-enriched proteins (Table 1) may contribute to further enrichment. It is unclear, however, whether α-synuclein occurs in SV clusters at concentrations that would enable it to undergo LLPS directly, without the intervention of other synaptic proteins. It must be noted that, while α-synuclein can undergo LLPS *in vitro*, this was performed in conditions of very high crowding, using 10% polyethyleneglycol (PEG), which may not represent the *in vivo* situation (Ray et al., 2020). This hypothesis is tempting, since proteins occupy ∼7–13% of the volume of synapses, close to the PEG levels used in the *in vitro* experiments (Wilhelm et al., 2014), but it is not clear whether the two situations are truly comparable. Importantly, despite its presumed high concentration in SV clusters, α-synuclein remains in a liquid state *in vivo*, at least in healthy neurons (Fortin et al., 2004; Unni et al., 2010). The fact that α-synuclein reversibly dissociates from SVs upon exocytosis (Fortin et al., 2005) indicates a robustness of its liquid state, even under conditions of dynamic turnover of SVs and proteins.

## A Dual View of α-Synuclein at the Synapse

The behavior of α-synuclein at the synapse can be viewed from two perspectives: 1) on the one hand, it may function as a supporter of the SV liquid phase thereby facilitating synaptic transmission; 2) on the other hand, the SV liquid phase may provide a “safe” environment that prevents its aggregation and misfolding. The factors contributing to the apparent stability of α-synuclein *in vivo* need to be elucidated, but the environment provided by the SV cluster is likely to be vital. This environment probably depends not only on the curved phospholipid surface of SVs but also on numerous integral SV proteins (Kim et al., 2021; Takamori et al., 2006) and soluble SV cluster proteins (Table 1). The role of the SV membrane in maintaining α-synuclein in a non-aggregated state is supported by *in vitro* data showing that acidic liposomes can prevent the maturation of liquid α-synuclein droplets into solid-like droplets (Figure 3B; [Hardenberg et al., 2021]). It can be assumed that the environment of the SV cluster has evolved in vertebrate phylogeny to simultaneously support neurotransmission and prevent the pathological transformation of its constituents.

## The Synaptic Vesicle Cluster Liquid Phase as a Possible Stabilizer of Non-Aggregated α-Synuclein

**FIGURE 4:**
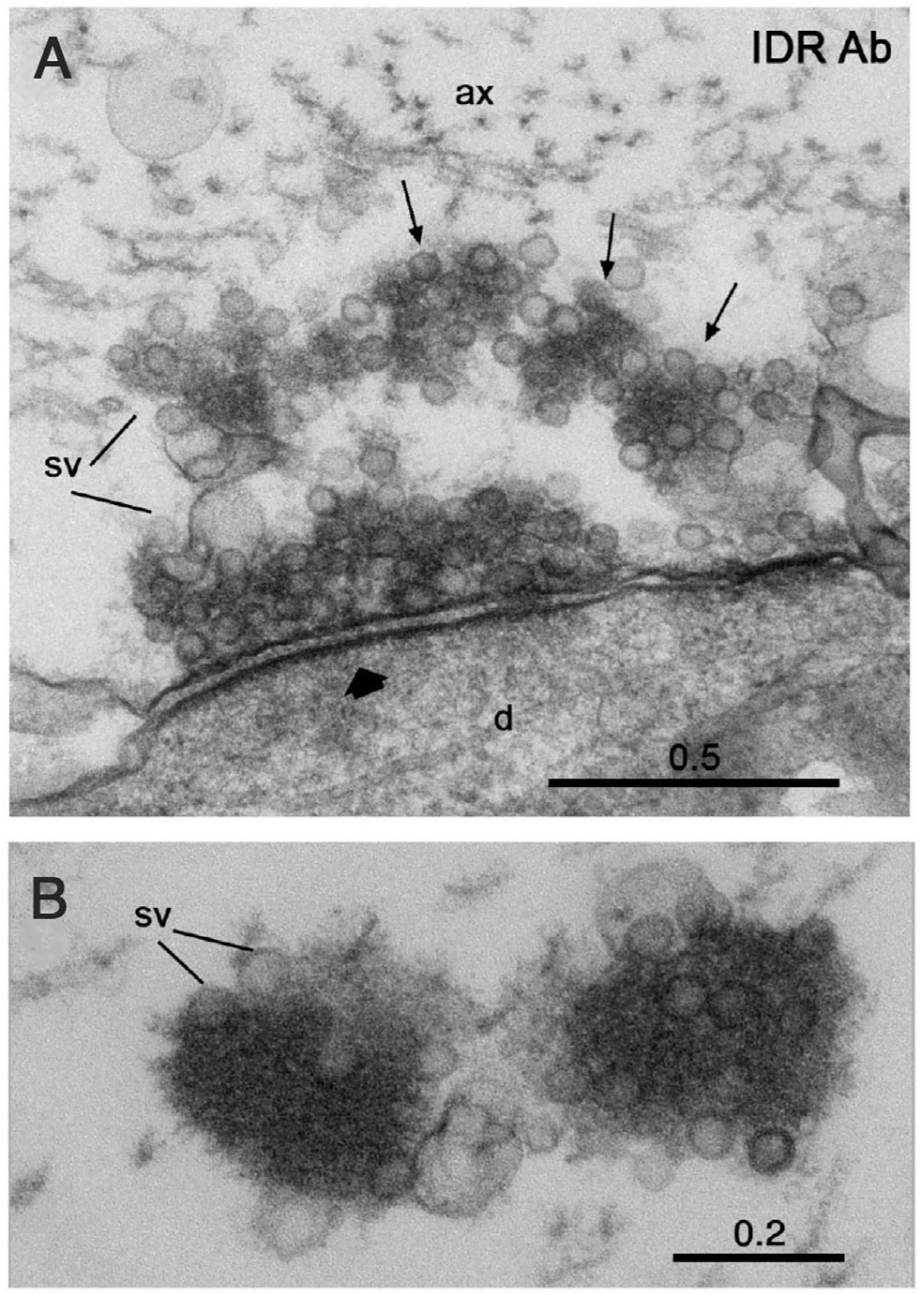
Disruption of the SV liquid phase by antibodies to the IDR of synapsin results in formation of electron-dense protein aggregates in the lamprey giant synapse. **(A)** Electron microscopic image showing partial disruption of the vesicle cluster and formation of electron dense condensates associated with synaptic vesicles (arrows) in a synapse in a reticulospinal axon at rest. **(B)** Electron dense condensates associated with synaptic vesicles (sv) in a synaptic region of a resting axon microinjected with antibodies at higher magnification. Scale bars in µm. From (Pechstein et al., 2020).

Several lines of evidence point to impaired synaptic proteostasis as one factor leading to protein aggregation in synucleinopathies (Nachman and Verstreken, 2021). We speculate that another contributing factor is a disturbance of the delicate SV cluster milieu. Such disturbance may be caused by elevated expression of α-synuclein. Indeed, multiplications of the gene locus encoding α-synuclein, SNCA, or mutations in upstream regulatory regions cause rare familial dominant PD (Runwal and Edwards, 2021; Sulzer and Edwards, 2019). Mutations within α-synuclein may also contribute. For example, the human A30P PD mutation impairs α-synuclein´s association with synapses and thus likely its binding to SVs (Fortin et al., 2005; Henning Jensen, 2001). An additional factor might be mutations in other proteins that contribute to the SV liquid phase. Parkinson-related mutations have indeed been found in some of the proteins listed in Table 1; (Nachman and Verstreken, 2021). Our recent *in situ* data are consistent with the general notion that the SV liquid phase is critical to prevent protein aggregation. Thus, acute disruption of the SV liquid phase and the associated vesicle pool in the lamprey reticulospinal synapse leads to the occurrence of electron-dense aggregates in the presynaptic region [Figure 4; (Pechstein et al., 2020)]. . It will now be of key interest to determine whether these aggregates contain misfolded proteins and how they are organized and evolve structurally in time.
